# Genomic Characterization and Phylogenetic Analysis of Linezolid-Resistant *Enterococcus* from the Nostrils of Healthy Hosts Identifies Zoonotic Transmission

**DOI:** 10.1007/s00284-024-03737-2

**Published:** 2024-06-14

**Authors:** Idris Nasir Abdullahi, Carmen Lozano, Myriam Zarazaga, Javier Latorre-Fernández, Søren Hallstrøm, Astrid Rasmussen, Marc Stegger, Carmen Torres

**Affiliations:** 1 Area of Biochemistry and Molecular Biology, OneHealth-UR Research Group, University of La Rioja, 26006 Logroño, Spain; 2 Department of Medical Laboratory Science, Faculty of Allied Health Sciences, College of Medical Sciences, Ahmadu Bello University, PMB 05 , Zaria, Nigeria; 3 Department of Bacteria, Parasites and Fungi, Statens Serum Institute, Copenhagen, Denmark; 4 Antimicrobial Resistance and Infectious Diseases Laboratory, Harry Butler Institute, Murdoch University, Perth, Australia

## Abstract

Linezolid resistance in *Enterococcus* spp. is increasingly considered critically important and a public health threat which mandates the need to understand their genomic contents and dissemination patterns. Here, we used whole-genome sequencing to characterize the resistome, virulome and mobile genetic elements of nine linezolid-resistant (LZD^R^) enterococci (seven *optrA*-*E. faecalis,* one *poxtA*-*E. faecium* and one *optrA*-*E. casseliflavus*) previously obtained from the nares of healthy dogs, pigs, pig farmers and tracheal samples of nestling storks in Spain. Also, the relatedness of the isolates with publicly available genomes was accessed by core-genome single nucleotide polymorphism (SNP) analysis. The *optrA* gene of the *E. faecalis* and *E. casseliflavus* isolates was located downstream of the *fexA* gene. The *optrA* gene in the *E. casseliflavus* isolate was carried in a plasmid (pURX4962), while those in the seven *E. faecalis* isolates were chromosomally located. The OptrA proteins were mostly variants of wild type (DP-2: Y176**D**/T481**P**; RDK: I104**R**/Y176**D**/E256K; DD-3: Y176**D**/G393**D;** and EDD: K3**E**/Y176**D**/G393**D**), except two that were wild type (one *E. faecalis* and one *E. casseliflavus*). The *poxtA* gene in the *E. faecium* isolate was found alone within its contig. The *cfrD* was upstream of *ermB* gene in the *E. casseliflavus* isolate and flanked by *IS*NCY and *IS*1216*.* All the LZD^R^ enterococci carried plasmid *rep* genes (2–3) containing tetracycline, chloramphenicol and aminoglycoside resistance genes. All isolates except *E. casseliflavus* carried at least one intact prophage, of which *E. faecalis*-ST330 (X4957) from a pig carried the highest (*n* = 5). Tn*6260* was associated with *lnuG* in *E. faecalis*-ST330 while Tn*554* was with *fexA* in *E. feaecalis-*ST59 isolates. All except *E. casseliflavus* (*n* = 0) carried at least two metal resistance genes (MRGs), of which *poxtA*-carrying *E. faecium*-ST1739 isolate contained the most (*arsA, copA, fief, ziaA, znuA, zosA, zupT,* and *zur*). SNP-based analyses identified closely related *optrA*-*E. faecalis* isolates from a pig and a pig farmer on the same farm (SNP = 4). Moreover, *optrA*- carrying *E. faecalis*-ST32, -ST59, and -ST474 isolates from pigs were related to those previously described from humans (sick and healthy) and cattle in Spain, Belgium, and Switzerland (SNP range 43–86). These findings strongly suggest the transmission of LZD^R^-*E. faecalis* between a pig and a pig farmer and potential inter-country dissemination. These highlight the need to strengthen molecular surveillance of LZD^R^ enterococci in all ecological niches and body parts to direct appropriate control strategies.

**Supplementary Information:**

The online version contains supplementary material available at 10.1007/s00284-024-03737-2.

## Introduction

Antimicrobial resistance (AMR) constitutes one of the major global health challenges that need a holistic “One Health” and “One World” approach to understand its emergence, evolution, and dissemination pattern [1]. Enterococci are very resilient as they can survive the effect of physicochemical agents for long periods [2]. *Enterococcus* is one of the suitable bacteria to study AMR across the “One Health” ecosystems, as certain species and genetic lineages could easily acquire or transfer critically important AMR to other species or genera in different hosts or niches via mobile genetic elements [3].

Linezolid (LZD) is one of the most important and “last resort” chemotherapeutic options for severe infections caused by vancomycin-resistant enterococci and multiresistant staphylococci [4]. Linezolid resistance (LZD^R^) is rare but represents a critically important public health problem that needs to be meticulously studied and reported at all levels. Linezolid is not used in animals but LZD^R^ could be selected by the use of other antibiotics, as is the case of phenicols or lincomycin, among others [5].

Linezolid resistance is mediated either by the acquisition of transferable genes (*optrA, poxtA,* and *cfr*), or due to mutations in the V domain of the 23S rRNA or amino acid changes in the ribosomal proteins L3, L4 and/or L22 of the enterococci [6–8].

Concerning the transferable LZD^R^ genes, *optrA* has been frequently reported [6]. At least 69 OptrA variants have been identified so far, with differences of 1–20 amino acid substitutions (i.e., of 97.1–99.8% amino acid identity) [8]. The *optrA* gene codes for an ATP-binding cassette F ribosomal protection protein that confers cross-resistance to chloramphenicol, linezolid and tedizolid [9]. The *poxtA* gene was identified and characterized in 2018 from a clinical MRSA in Italy [10]. This gene shares about 32% nucleotide homology with *optrA* and uses a similar mechanism to confer resistance to linezolid and exists in two variants, *poxtA* and *poxtA2* [10, 11].

The *cfr* gene encodes a methyltransferase that modifies position A2503 of the 23S rRNA and confers cross-resistance to streptogramin A, lincosamides, pleuromutilins, phenicols and linezolid [8, 12]. There are four variants of the *cfr* gene, i.e., *cfrB, cfrC, cfrD,* and *cfrE*, of which *cfrB* and *cfrD* do not confer resistance on their own, except in combination with other linezolid resistance genes [13, 14]. Moreover, the *cfrC* and *cfrE* have not been reported in any *Enterococcus* spp. [6]. It appears that *cfr*-mediated LZD^R^ is predominantly found in staphylococci whereas the *cfr*-like gene, *cfrD,* is common in enterococci [6].

The mutation-mediated LZD^R^ enterococci emerged following linezolid chemotherapy in clinical practice with the predominant being the G2576T or G2505A in 23S rRNA [15]. However, other mutations leading to high-level linezolid resistance such as G2447U, G2576U, and G2504A, among others, have also been reported in enterococci [14]. It is important to remark that LZD^R^ due to target mutations is not transferable as they are not bound to mobile genetic elements. However, the acquired LZD^R^ genes are often carried by plasmids while in some instances, they are chromosomally located [6].

Linezolid-resistant enterococci and the mechanisms of resistance involved have been reported in Spain [7, 16–22]. However, few reports on their complete genomic characterization have been published [18, 23] and none on healthy human and animal populations. As the incidence of LZD^R^ enterococci is gradually increasing all over the world, it is important to mount a genome-based surveillance system to track their dissemination patterns and relatedness with other reported cases from other countries. The present study characterized the genomic profile in a collection of enterococcal isolates belonging to three species (*E. faecalis, E. faecium* and *E. casseliflavus*) carrying LZD^R^ genes previously identified from healthy animals and humans [7], to characterize their resistomes, virulomes, mobile genetic elements and to determine their phylogenetic relatedness with other publicly available strains with similar sequence types and AMR genes.

## Materials and Methods

Nine LZD^R^ enterococci were obtained in a previous study [7], and were included in the present work for complete genomic characterization. These isolates were all of nasal origin (except one of tracheal origin in stork), and belonged to three species: a) seven *optrA*-carrying *E. faecalis* isolates of pigs (*n* = 5), pig farmers (*n* = 1) and dogs (*n* = 1); b) one *optrA/cfrD*-carrying *E. casseliflavus* isolate from a pig; and c) one *poxtA*-carrying *E. faecium* isolate of a stork (Table 1). The *E. faecalis* isolates from the pig farms were selected based on their origins, sequence types (STs) and AMR genes. Details on the strategies of sample collection, processing, isolation, identification of bacteria, their antibiogram and AMR genotyping were presented in our previous study [7].

**Table 1:** Genomic characteristics of 9 linezolid-resistant *Enterococcus* spp. investigated in this study

Isolate ID^a^		Source/ ID	ST/ CC	No. of contigs/ Genome size (Mb)	LZD^R^ genes in chromosome (Amino acid changes on LZD^R^ protein)	LZD^R^ genes in plasmids	LZD MIC (μg/ml)^b^	other AMR genes	Metal resistance genes	Chromosomal point mutations	Prophage	Transposons	Plasmid *rep*licons (associated AMR^a^ genes)	Insertion sequence	Virulence factors
*E. casseliflavus* X4962		Pig/Farm A -P1	NT	247/3.7	*cfrD* (wild type)	*optrA* [wild type (by *rep*US40)	8	*fexA, lnuB, lsaE, aph3’, dfrG, vanC3*	None	None	None	None	*rep*1, *rep*14b, *rep*US1, *rep*US41	ISEnfa1	None
*E. faecium* X3877		Stork/ Landfill/	ST1739	136/2.6	*poxtA* (wild type)	None	8	*fexB*	*arsA, copA, fief, ziaA, zosA, zupT, zur, znuA*	^c^17 mutations in PBP5	vB_IME197 BCJA1c	None	*rep*1, *rep*29, *rep*US15	ISSsu5	*acm, efaAfm*
*E. faecalis* X4957		Pig/ farm A -P8	ST330	164/3.1	*optrA* (RDK: I104**R**, Y176**D**, E256**K)**	None	10	*cat, fexA, lnuG, lsaA, ermA, tet*(L)*, tet*(M)*, aac6’-aph2″, aph3’, dfrG*	*cutC, tcrB, znuA*	ParC (S80I), GyrA (E87G)	phiEf11 phiFL4A phiFL2A EFC _1 LP_101	Tn*6260* (*lnuG*)	*rep1*, *rep9a* (*tet*(L)*, tet*(M)*, cat*), *repUS1, repUS43* (*tet*(L)*, tet*(M)*, cat*)	ISEnfa1	*ace, agg, cad, camE, cOB1, cCF10, ebpA, ebpC, elrA, efaAfs, fsrB, gelE, hylA, hylB, srtA, tpx*
*E. faecalis* X5386		Pig/ farm B-P1	ST330	137/3.0	*optrA* (DP-2: Y176**D**, T481**P**)	None	14	*cat, fexA, lnuG, lsaA, ermA, tet*(L)*, tet*(M)*, aac6’-aph2″, ant4’, aph3’, dfrG*	*cutC, znuA*	ParC (S80I), GyrA (E87G)	phiEf11 BCJA1c phiFL4A	Tn*6260* (*lnuG*)	*rep9a* (*tet*(L)*, tet*(M)*, cat*)*, rep9b, rep9c, repUS43* (*tet*(L)*, tet*(M)*, cat*)*, repUS52* (*ant4’*)	None	*ace, agg, camE, cOB1, cCF10, dad, ebpA, ebpC, elrA, efaAfs, fsrB, gelE, hylA, hylB, SrtA, tpx*
X5463		Pig farmer/ farm B- F2	ST330	116/2.9	*optrA* (DP-2: Y176**D**, T481**P**)	None	10	*cat, fexA, lnuG, lsaA, ermA, tet*(L)*, tet*(M)*, aac6’-aph2″, ant6’, aph3’, dfrG*	*cutC, tcrB, znuA*	ParC (S80I), GyrA (E87G)	phiFL4A Lj928 BCJA1c	Tn*6260* (*lnuG*)	*rep9a* (*tet*(L)*, tet*(M)*, cat*)*, rep9b, repUS43* (*tet*(L)*, tet*(M)*, cat*)	ISS1N	*ace, agg, cad, camE, cOB1, cCF10, dad, ebpA, ebpC, elrA, efaAfs, fsrB, gelE, hylA, hylB, srtA, tpx*
*E. faecalis* X5799		Pig/ Farm D-P5	ST59	139/ 2.9	*optrA* (wild type)	None	2	*cat, fexA, lnuB, lsaA, lsaE, ermA, tet*(L)*, tet*(M)*, aac6’-aph2″, aph3’, dfrG*	*cutC, tcrB, znuA*	None	phiFL4A	Tn554 (*fexA, optrA*)	*rep9a, rep9c, repUS43*	ISS1N, ISEnfa4	*ace, agg, camE, cOB1, cCF10, cylA, dad, ebpA, ebpC, elrA, efaAfs, fsrB, gelE, hylA, hylB, srtA, tpx*
*E. faecalis* X5809		Pig/ Farm D-P10	ST474	100/2.8	*optrA* (EDD: K3**E**, Y176**D**, G393**D**)	None	12	*cat, fexA, lnuB, lsaA, lsaE, ermA, tet*(L)*, tet*(M)*, aac6’-aph2″, aph3’, dfrG*	*cutC, tcrB, znuA*	ParC (S80I), GyrA (E87G)	phiFL3A	None	*rep9a* (*tet*(L)*, tet*(M)*, cat*)*, rep9c, repUS43* (*tet*(L)*, tet*(M)*, cat*)	ISS1N	*ace, agg, camE, cOB1, cCF10, cylA, dad, ebpA, ebpC, elrA, efaAfs, fsrB, gelE, hylA, hylB, srtA, tpx*
*E. faecalis* X5445		Pig/ farm B-P8	ST32	122/2.9	*optrA* (DD_3: Y176**D**, G393**D**)	None	10	*cat, fexA, lnuG, lsaA, ermA, ermB, tet*(L)*, tet*(M)*, aac6’-aph2″, ant9’, aph3’, dfrG*	*cutC, tcrB, znuA*	None	phiFL3A EFC_1	None	*rep9a* (*tet*(L)*, tet*(M)*, cat*)*, rep9c, repUS43* (*tet*(L)*, tet*(M)*, cat*)	ISS1N	*ace, agg, cad, camE, cOB1, cCF10, cylA, dad, ebpA, ebpC, elrA, efaAfs, fsrB, gelE, hylA, hylB, srtA, tpx*
*E. faecalis* X6347		Dog/ household 56	ST585/ CC5	148/2.8	*optrA* (DP-2: Y176**D**, T481**P**)	None	12	*fexA, lnuB, lsaA, ermA, ermB, tet*(L)*, tet*(M)*, str*	*cutC, tcrB, znuA*	ParC (S80I)	PHBC6A5A	None	*rep7a* (*str*), *rep9a* (*tet*(L)*, tet*(M)*, cat*)*, rep9b, repUS43* (*tet*(L)*, tet*(M)*, cat*)	None	*ace, agg, cad, camE, cOB1, cCF10, cylA, cylL dad, ebpA, ebpC, elrA, efaAfs, fsrB, gelE, hylA, hylB, srtA, tpx*

*AMR* Antimicrobial Resistance, *F* pig farmer, *P* pig, *ST* Sequence type, *CC* Clonal complex

^a^All strains were of nasal origin, except *E. faecium* X3877 which was from a tracheal sample

^b^MIC to linezolid [7]

^c^17 mutations in PBP5 = S27G, A68T, A216S, T172A, V24A, 885D, K144Q, A499T, L177I, N496K, G66E, E100Q, D204G, P667S, E525D, T324A, R34Q]

### Whole Genome Sequencing, Assembly, and Phylogenetic Analyses

Whole genome sequencing of the selected LZD^R^ enterococci was carried out on the NextSeq 550 platform (Illumina), while the *E. casseliflavius* isolate was further sequenced on the MinION platform (Oxford Nanopore Technologies (ONT), Oxford, United Kingdom) as described here. Single colonies were obtained from a fresh over-night blood agar plating and resuspended in enzymatic lysis buffer [Proteinase K (Roche); Lysozyme (Sigma)] and incubated at 37 °C (30 min) and 55 °C (1 h). The final buffer composition was as follows: 1× phosphate buffered Saline (pH 7.2 (ThermoFisher Scientific), 10× solution diluted to 1× in nuclease-free water), 20 mM Tris–HCl (pH 8; ThermoFisher Scientific), 2 mM EDTA (ThermoFisher Scientific), 1.2% Triton X-100 (Merck), 1.7 mg/ml Proteinase K (Roche), 20 mg/ml Lysozyme (Sigma).

The MagNA Pure 96 DNA and Viral NA Small Volume Multi-Sample Kit (Roche) was used to extract genomic DNA according to the manufacturer's instructions. DNA was quantified using the Quant-iT dsDNA BR and HS Assay Kits (Thermo Fisher Scientific, Scoresby, VIC, Australia) and fluorescence was measured on a FLUOstar Omega (BMG LabTech). For short-read sequencing on the Illumina platform, sequencing libraries were prepared using the Illumina Nextera XT DNA Library Preparation Kit (Illumina). The final libraries were analyzed on a TapeStation 4200 (Agilent) before sequencing on the NextSeq 550 platform (Illumina) using a 300-cycle kit to obtain paired-end 150 bp reads, as previously described [24]. For long-read sequencing on the MinION platform, the ONT rapid barcoding kit (SQK-RBK110.96) was used to generate the sequencing library. Four samples were multiplexed and sequenced on an R9.4.1 flow cell. Reads were demultiplexed and base-called with the base-calling model Guppy (v.6.1.5) at super-accurate setting and filtered on quality score > 10. All the genomes analyzed in this study were de novo assembled using SPAdes (v.3.15.5), performing the in silico typing with the settings of a minimum of 90% coverage and 80% identity. Core-genome single nucleotide polymorphisms (SNPs) were detected with the NASP pipeline v.1.0.0 [25]. GATK (v.4.2.2) was used to call SNPs and excluded positions featuring < 90% unambiguous variant calls and < 10 depth. IQ-TREE (v.2.1.2), was used to construct the phylogenetic trees using ModelFinder with 100 bootstraps. The graphical data were added to the phylogenies with iTOL v.6.6 [26].

### Genome Annotation, Typing and In Silico Analysis

The STs were determined with MLST (v.2.16, https://cge.food.dtu.dk/services/MLST/). Virulence factors, plasmid replicons, and antimicrobial resistance genes were identified using ABRicate (v.0.9.0) [27], and the respective databases VFDB [28], Plasmidfinder [29], and Resfinder [30] from the Center for Genomic Epidemiology. Mutations associated with AMR were identified using ResFinder (v4.1) [30] and PointFinder [31]. Phaster was used to identify all prophage elements [32]. The genetic environments of *optrA, poxtA* and *cfrD* genes were illustrated using the reference strains [*E. faecalis* (GenBank accession ID KP399637), *E. faecium* plasmid pGZ8 (GenBank accession ID CP038162), *E. faecium* (GenBank accession ID MN831413) and *E. faecalis* (GenBank accession ID CP097040)] in EasyFig Software**.** The OptrA variants based on amino acid substitutions deduced from the *optrA* sequences, were analyzed according to a previously described nomenclature [8], using WP_063854496.1 as the wild-type reference.

### Phylogenetic Analysis by Core-Genome Single Nucleotide Polymorphism

The SNPs between the genomes of our seven *optrA*-*E. faecalis* isolates and those of 12 publicly available *optrA*-positive *E. faecalis* isolates was analyzed (GenBank accession numbers: SRR17662732, ERR2008110, ERR2008112, ERR1599987, ERR1599986, ERR2008113, ERR2008114, SRS7549315, SRS7549355, SRS7549357, SRS7549371, SRS7549400). These 12 genomes were selected because they carried the *optrA* gene and belonged to the same genetic lineages as the seven *E. faecalis* strains from this study.

### Genome Availability

All the raw genome reads of the LZD^R^ enterococci have been deposited at the European Nucleotide Archive under Study Accession number PRJEB62654. The *optrA*-associated plasmid in *E. casseliflavus* (pURX4962) was deposited in GenBank with the accession number OR069652.

## Results

### Genome Features

The genomes of the seven LZD^R^ *E. faecalis* isolates had sizes in the range of 2.8–3.1 Mb and contigs range from 100 to 164 (Table 1). The LZD^R^ *E. faecium* isolate had a genome size of 2.7 Mb and 136 contigs, whereas the LZD^R^ *E. casseliflavus* isolate had a genome size of 3.7 Mb and 247 contigs (Table 1).

### Antimicrobial and Metal Resistance

The minimum inhibition concentrations (MICs) of all enterococci to linezolid ranged from 2 to 14 μg/ml **(**Table 1**).** The in silico analysis of the *optrA* sequences of the eight *E. faecalis*/*E.casseliflavus* isolates revealed that two of them harbored the gene of the wild-type OptrA (*E. faecalis* and *E. casseliflavus,* both of pig origin), while the remaining six *E. faecalis* isolates carried the gene of four OptrA variants (DP-2: Y176**D**/T481**P**; RDK: I104**R**/Y176**D**/E256**K**; DD-3: Y176**D**/G393**D**; and EDD: K3**E**/Y176**D**/G393**D**) (Table 1). The OptrA variant DP-2 was detected in three *E. faecalis* isolates of pig, pig farmer and dog origin (ST330 and ST585), and the OptrA variants RDK, DD-3, and EDD in three *E. faecalis* isolates recovered from pigs (Table 1). Also, the LZD^R^-*E. faecium* isolate carried a *poxtA* type 1 gene (Table 1). In addition, all the *optrA*-positive enterococci co-carried the *fexA* gene; the *fexB* gene was co-carried by the *poxtA*-positive *E. faecium* isolate. Moreover, the *E. casseliflavus* co-carried the *cfrD* gene.

Beside the AMR genes presented in our previous studies [7], the following genes were detected: (a) *E. casseliflavus* isolate (X4962) carried the *dfrG, vanC3, lnuB, aph3’* and *lsaE* genes; (b) *E. faecalis*-ST330 (X5386 and X5809) from pigs of farms B-D carried the *ant4’, dfrG, lsaA,* and *aph3’*genes; (c) *E. faecalis-*ST59 (X5799) carried the *dfrG, lnuB, lsaE,* and *lsaA* genes*;* (d) *E. faecalis*-ST32* (*X5445*)* from a pig of farm B carried the *lsaA, ant9’, dfrG, lnuG,* and *aph3’*genes; (e) *E. faecalis-*ST330 (X5463) from a pig farmer of farm B carried the *ant4’, dfrG, lsaA,* and *aph3’*genes; (f) *E. faecalis-*ST585 (X6347) from a dog carried the *lnuB* and *lsaA* genes*.* There were no additional AMR genes detected in the *E. faecium*-ST1739 isolate (X3877) from a nesting of stork foraging in landfills (Table 1).

Aside from the AMR genes, chromosomal point mutations leading to ciprofloxacin resistance were detected in *E. faecalis*-ST330 isolates from pigs of farms B and D, associated with amino acid changes in ParC (S80I) and GyrA proteins (E87G). Moreover, only one amino acid change in ParC (S80I) was detected in the dog *E. faecalis*-ST475 isolate. In addition, 17 different amino acids substitutions were identified on the penicillin-binding protein 5 (S27G, A68T, A216S, T172A, V24A, 885D, K144Q, A499T, L177I, N496K, G66E, E100Q, D204G, P667S, E525D, T324A, R34Q) of the *E. faecium* isolate from stork nestling (*poxt*A-positive), some of them leading to penicillin resistance (Table 1).

All LZD^R^ isolates except *E. casseliflavus* X4962 carried at least two metal resistance genes (MRGs), of which *E. faecium*-ST1739 carried most of them (*arsA, copA, fief, ziaA, znuA, zosA, zupT, zur)* (Table 1)*.* Moreover, *E. faecalis* X5386 carried only two of the MRGs (*cutC* and *znuA*).

### Virulence Determinants

Many virulence genes that have been associated with surface adherence, biofilm formation, and cytolysis were detected in the *optrA*-carrying *E. faecalis* isolates, most frequently being the *ebpA, tpx, elrA, hylA, srtA, gelE, fsrB, ace, cOB1, cCF10, dad, agg, camE, efaAfs, hylB,* and *cylA* genes. In the *E. faecium* X3877 isolate, only *acm* and *efaAfm* genes were found. However, none of these genes were identified in the *E. casseliflavus* X4962 isolate (Table 1).

### Mobile Genetic Elements

**Fig. 1:**
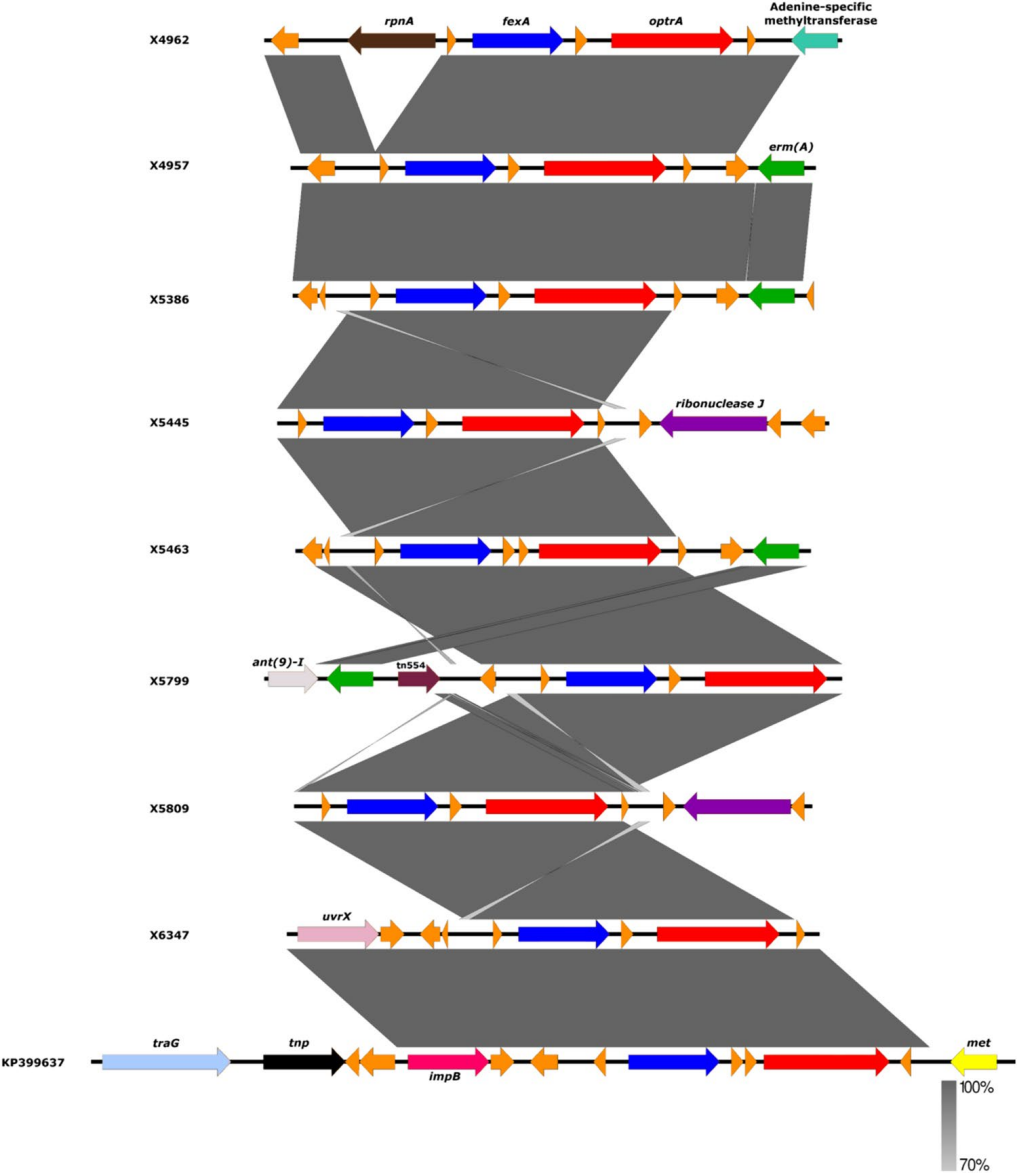
Genetic environment of the *optrA* gene in the eight *E. faecalis* and *E. casseliflavus* isolates from healthy pigs, pig farmer, and a dog. Shown in the figure are AMR genes located in the same contigs and frames with their corresponding mobile genetic elements. The percentage of identity and scale bar legends are presented on the right side of the image. The comparison was made with a reference *E. faecalis* strain E349 (GenBank Accession number: KP399637) (colour figure online)

All the enterococci carried at least one plasmid replicon gene (1–5 *rep* genes), however, only some of the plasmid replicons (2–3 per isolate) were associated with AMR genes. The three *optrA*-positive *E. faecalis*-ST330 isolates carried different number of plasmid replicons (Table 1). The *repUS52* in strain X5386 was found co-located with the aminoglycoside resistance *ant4’* gene. Three resistance genes (*tet*(L)*, tet*(M)*,* and *cat*) were bound on plasmid *rep9a* and *rep*US43 of all *E. faecalis* isolates from the pigs and pig farmers **(**Table 1**).** The aminoglycoside resistance gene, *str* was found on plasmid *rep7* in the *E. faecalis* isolate from a dog (X6347). Moreover, the *agg* virulence gene was also co-located on the *rep*9a contig of *E. faecalis*-ST330 (X5463) and *E. faecalis*-ST32 (X5445). Three replicons were identified in the *poxtA*-carrying *E. faecium* isolate (*rep*29, *rep*1, *rep*US15) and none was co-located with any other AMR genes. Moreover, five replicons (*rep14b, repUS41, repUS1, rep1*, and *rep40*) were identified in the *optrA*-positive *E. casseliflavus* X4962 isolate, of which *repUS40* was associated with the *optrA-fexA* genes (Fig. 1), and was 99.83% identical with plasmid *pE3954* of *E. faecalis* (GenBank accession no: KP399637).

Two different transposons were identified, viz: Tn*6260* and Tn*554* associated with *lnuG* and *fexA* genes of *E. faecalis*-ST330 and -ST59 isolates, respectively. Also, the Tn*6260* identified in three of our *optrA*-positive *E. faecalis* isolates have previously been shown associated with *lnuG*.

**Fig. 2:**
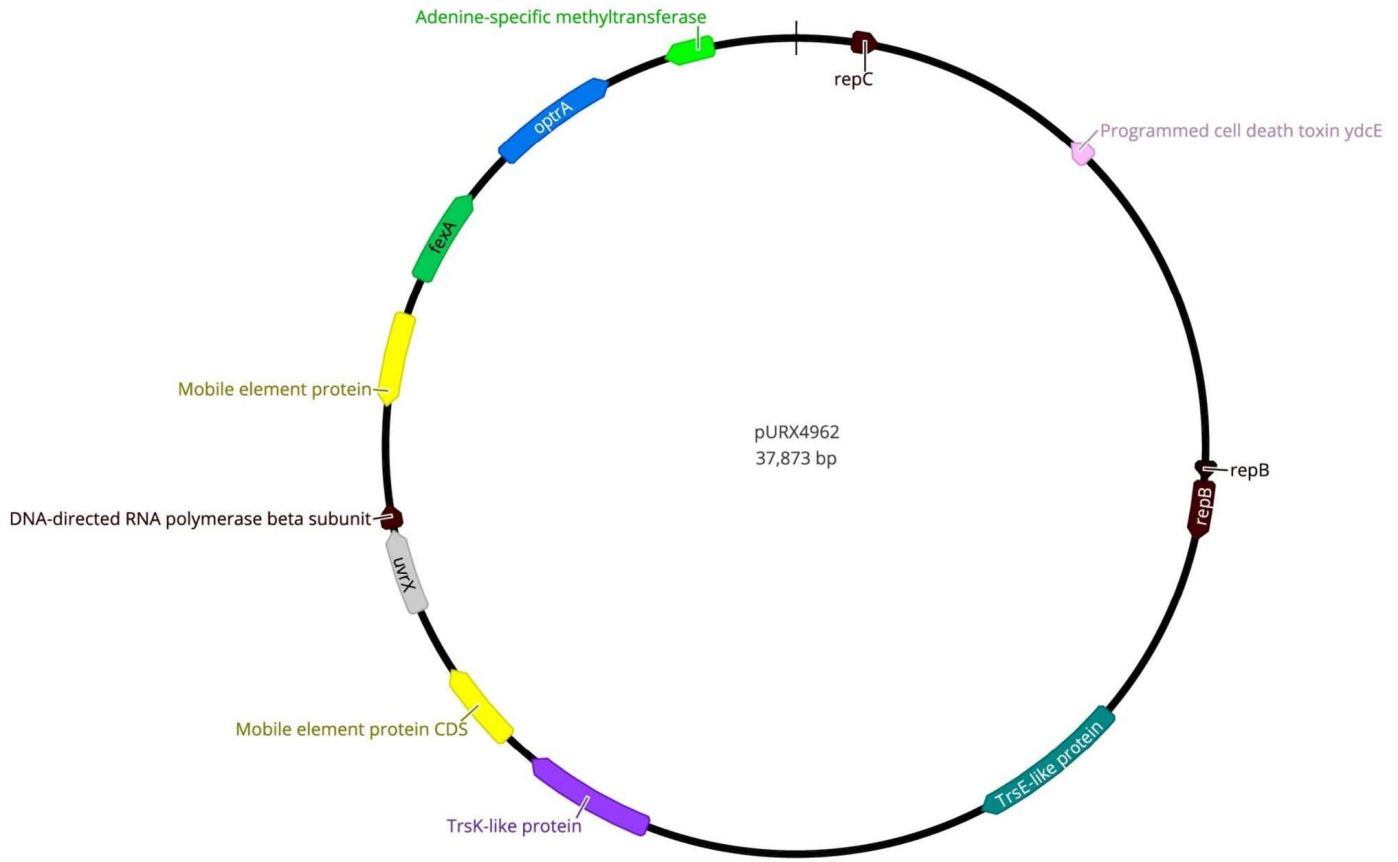
Genetic representation of the *optrA*-carrying plasmid pURX4962. Colors and arrows indicate the represented genes and their orientation (color figure online)

Different insertion sequences were detected among our LZD^R^ isolates: *E. faecalis*-ST59 (ISS1N and ISEnfa4), *E. faecalis*-ST330 (ISEnfa1), *E. faecium* (ISSsu5) and *E. casseliflavus* (ISEnfa1) (Table 1). The *optrA* gene was chromosomally located in all our *E. faecalis* isolates. Nevertheless, the *optrA* gene of *E. casseliflavus* strain X4962 was located in a plasmid (37.9 kb, pURX4962) (Fig. 2), that showed 99.98% similarity with the one of an *E. faecalis* strain from China (GenBank Accession number: KP399637.1). All our LZD^R^ *E. faecalis* and *E. faecium* isolates carried at least one prophage, of which *E. faecalis* -ST330 (X4957) from a pig carried the highest variety (*n* = 5), viz: phiEf11, phiFL4A, phiFL2A, EFC_1 and LP_101 **(**Table 1**)**.

### Genetic Environment of the Linezolid Resistance Genes

**Fig. 3:**
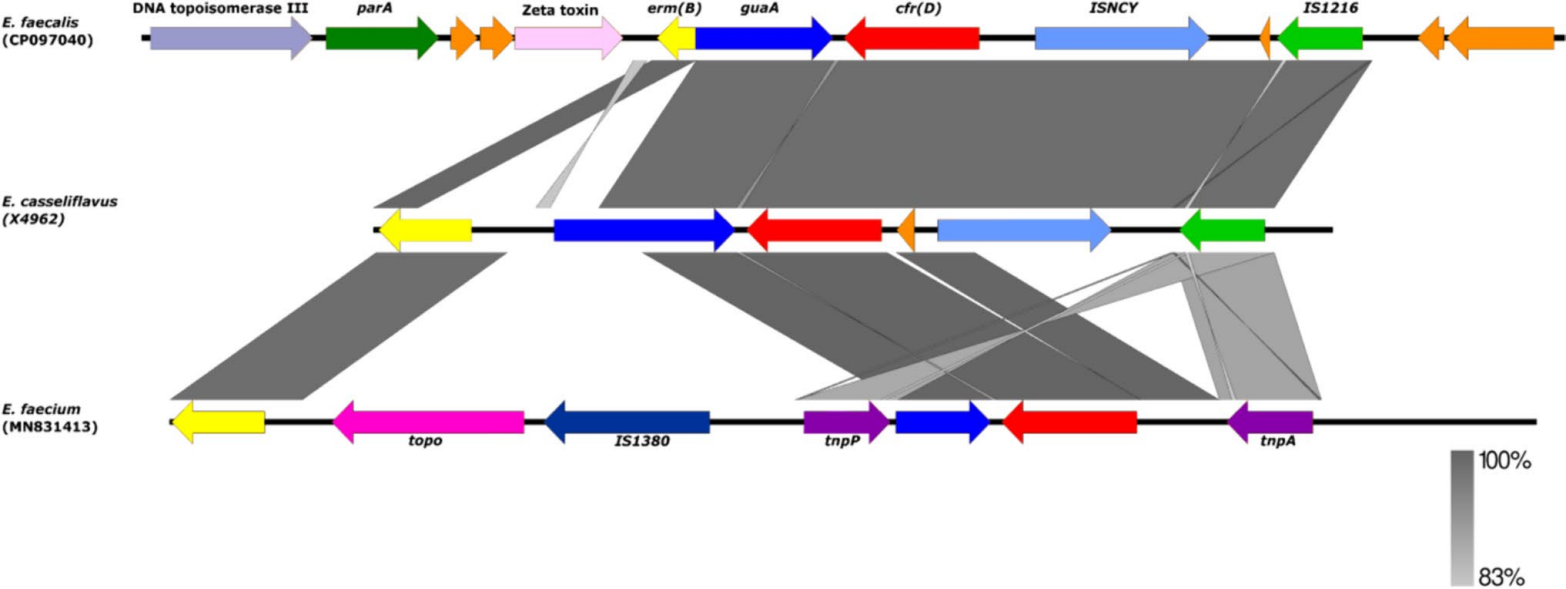
Schematic comparison between the environment of the *cfrD* gene in the *E. casseliflavus* isolate X4962 with *E. faecalis* (GenBank Accession number: CP097040) and *E. faecium* (GenBank Accession number: MN831413) (color figure online)

Five different genetic environments of the *optrA* gene were detected among the eight isolates that carried this gene: (a) *E. casseliflavus* X4962 (OptrA wild type); (b) *E. faecalis* X4957, X5386, and X5463 (OptrA—variants RDK and DP-2); (c) *E. faecalis* X5445 and X5809 (OptrA -variants DD-3 and EDD); (d) *E. faecalis* X5799 (OptrA- wild type); and (e) *E. faecalis* X6347 (OptrA variant DP-2). The *fexA* gene, which confers resistance to phenicols, was detected upstream of the *optrA* gene in all the eight *E. faecalis* and *E. casselifavus* isolates (Fig. 1). Moreover, an *ermA*-like gene was detected in the seven *optrA*-carrying *E. faecalis* isolates. Of these, the *ermA*-like gene was found in the environment of the *optrA* gene in four of isolates (Fig. 1). This *ermA*-like gene was identical to that detected in *Streptococcus suis* (GenBank accession number: EU348758). Regarding the *cfrD* gene, we could identify the presence of a *guaA* gene encoding a glutamine-hydrolyzing guanosine monophosphate synthase in the downstream region. Upstream of the *cfrD* gene, we detected the *ermB* gene flanked by IS1216 and ISNCY (Fig. 3)*.* The genetic environment of the *cfrD* gene (1074 bp) revealed 100% nucleotide similarity with that of an *E. faecium* isolate in France (GenBank accession number: NG_067192).

**Fig. 4:**
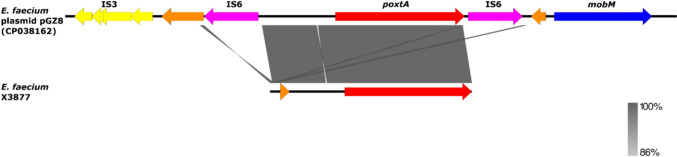
Genetic environment of the *poxtA* gene in the *E. faecium* isolate X3877 in comparison with the previously described plasmid-bound *poxtA* gene in the *E. faecium* strain pGZ8 (GenBank Accession number: CP038162) (color figure online)

Sequence analysis revealed that *E. faecium*-X3877 harbored the wild-type *poxtA* gene with 100% nucleotide sequence identity to that of *E. faecium* plasmid pGZ8 (GenBank accession number: CP038162) (Fig. 4).

### Relatedness of the *E. faecalis* Isolates

First, SNP analyses identified high relatedness (SNP = 4) of a pig isolate (X5386) with that of a pig farmer (X5463) from the same farm in our study **(**Supplementary Table S1**).** Both isolates carried the gene associated with the same OptrA variant (DP-2) and the same *optrA*-genetic environment and were *E. faecalis* of the lineage ST330.

**Fig. 5:**
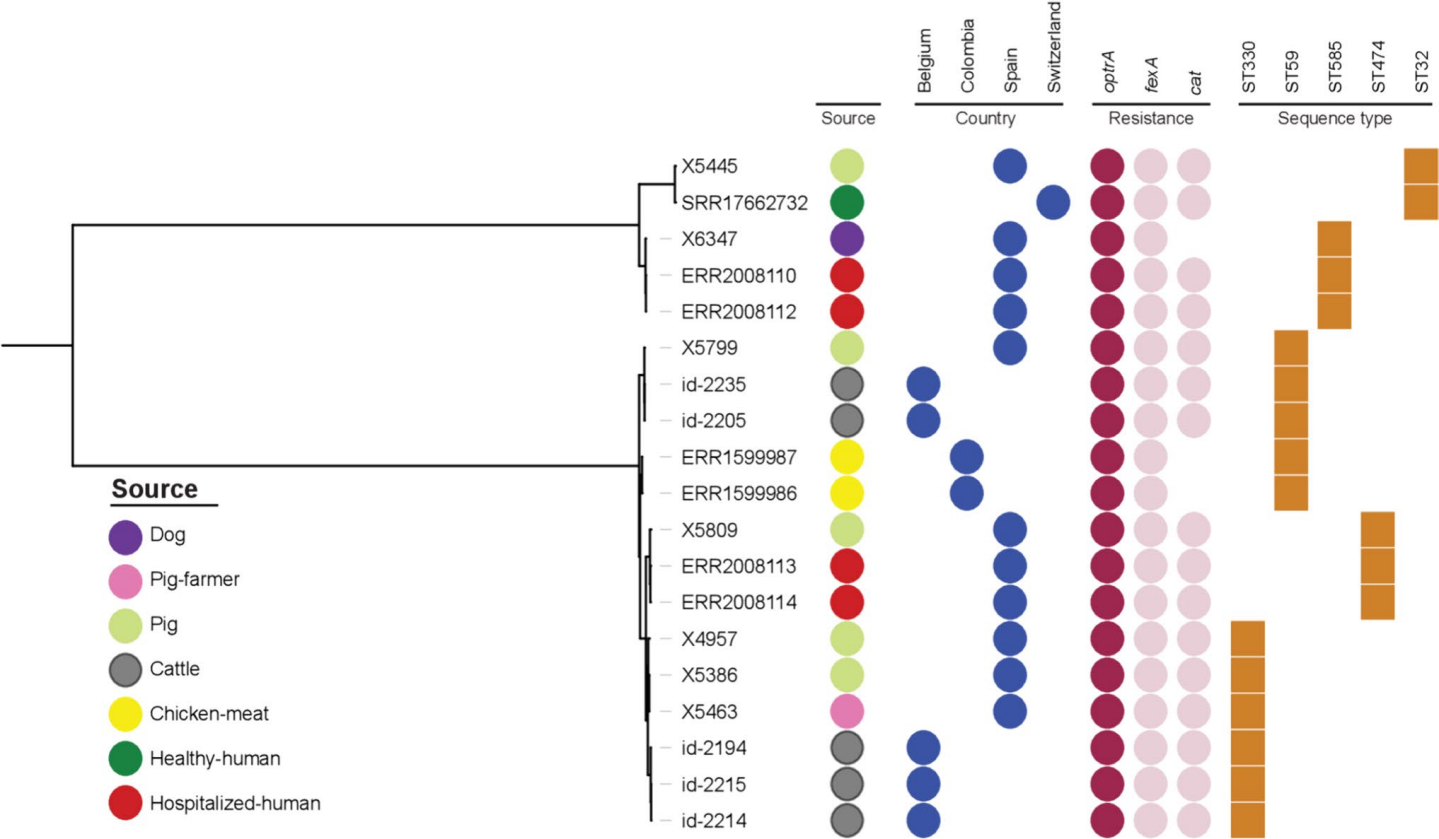
Phylogenetic tree based on core-genome SNPs analysis of seven *optrA*-carrying *E. faecalis* isolates from healthy hosts in this study with twelve publicly available *E. faecalis* genomes with similar STs and linezolid resistance genes. Colors (in circles) of the AMR genes are as follows: dark purple for *optrA* while and light purple for *fexA* and *cat* (color figure online)

Then, analyses with other publicly available genomes revealed relatedness of *E. faecali*s-ST32 (X5445) that was closely related to an isolate from a healthy human (SNP = 86) in Switzerland (SRR17662732). Moreover, the pig isolate (X5799) was related to two isolates from cattle origin (SNP = 44 and 48) in Belgium (id2205 and id2235) **(**Fig. 5** and **Supplementary Table S1**).** Furthermore, the pig isolate (X5809) was closely related (< 50 SNP) to two isolates previously described from hospitalized patients in Spain (ERR2008113 and ERR2008114).

## Discussion

Here we present a genomic investigation of nine LZD^R^ enterococci from four different healthy host types (humans, pigs, dogs, and storks). To the best of our knowledge, this is the first genomic comparative study on LZD^R^ enterococci in healthy human and animal populations in Spain.

Since the first detection of LZD^R^ enterococci in 2003 in Spain, two years after the approval of linezolid for clinical use [22], the detection rate of LZD^R^ has risen significantly, especially during and after the post-COVID-19 era [32]. In our previous study [7], LZD^R^ genes were detected after the selective inclusion of chloramphenicol resistance phenotype and characteristic green colonies on CHROMagar LIN by the three *Enterococcus* species (*E. faecalis*, *E. faecium* and *E. casseliflavus*). This was not expected as the animals and human hosts were healthy and were not on any antibiotic chemotherapy for at least six months before their enrolment into the study. This suggests that LZD^R^ could persist for a long period in enterococci, considering that the bacterial genus is very tolerant to environmental factors such as sunlight and temperature [2].

In a recent study conducted by our research group and others in Spain, LZD^R^ genes were detected from hospital settings with a high frequency of the *optrA*/*poxtA* genes among *E. faecalis* and *E. faecium* [17, 18]. This partly corroborated our finding as all the *E. faecalis* carried the *optrA* gene, but not *poxtA*, as in the case of Ruiz-Ripa et al. [18].

Worth mentioning is that most of the *optrA* genes carried by the *E. faecalis* strains from this current study (6/7) corresponded to variants of the OptrA wild-type (DP-2, RDK, DD-3, and EDD). The OptrA variants may affect the level of MIC value for linezolid [8, 33, 34], in which some strains show low MICs while others are within the breakpoint classified as resistance. In a study that showed a correlation between linezolid MIC and OptrA variants*,* it was detected that the EDM variant was associated with linezolid MICs ≤ 4 μg/ml, while the variant RDK (also found among our enterococci) with MIC values ≥ 8 μg/ml [34]. In the present study, all the LZD^R^ enterococci had MICs of ≥ 8 µg/ml, except one isolate carrying a wild-type OptrA (X5799) with an MIC of 2 μg/ml. Nevertheless, this unique finding implies that LZD^R^ may go unnoticed by phenotypic assays unless nucleic acid amplification tests are performed. As indicated in our previous study [7], chloramphenicol resistance could be considered as a marker to screen for linezolid resistance genes. Nevertheless, genes that mediate multidrug resistance (MDR) phenotypes identified from our isolates are frequent findings in LZD^R^ enterococci from previous studies [16–20]. Interestingly, none of the isolates was resistant to other last-resort antibiotics such as vancomycin, which could serve as an alternative for the LZD^R^ enterococci in clinical practice.

It is important to remark that the classification of *optrA* variants and their nomenclature is not uniform. In this regard, two types of classifications are available; one is based on amino acid changes in the wild-type OptrA protein (the one used in this study) while the other is based on numerical classification of the variants as a result of mutations in the nucleotides of the *optrA* gene [8, 35].

*Enterococcus faecium* showing ampicillin resistance is an additional problem in the clinical chemotherapy of enterococci [36]. Chromosomal point mutations putatively conferring resistance to linezolid (in 23S rRNA and ribosomal proteins L3/L4/L22) were not detected in our isolates. However, *E. faecalis*-ST330 from healthy pigs and pig farmer presented ParC (S80I) and GyrA (E87G) anino acid changes associated with fluoroquinolone resistance, which are commonly identified in samples obtained from livestock, foodstuffs, and from human infections [37, 38].

Several MRGs were identified in most of the isolates from this study. Specifically, *cutC* and *znuA* encode for copper homeostasis protein and high-affinity zinc uptake binding protein ZnuA, respectively [39]. Other LZD^R^ isolates carried three MRGs, *cutC, tcrB,* and *znuA*. It is important to highlight that metal resistance is a matter of public health concern due to its potential hazards in the food chain and co-selection of other AMRs [40]. Specifically, MRGs are common in enterococci strains due to the frequent use of some metals, such as zinc micronutrients in livestock feed supplements [41]. It is important to emphasize that the feed-relate source of these metals could have been deposited in the environment which caused the bacteria to develop resistance to these metals.

More worrisome is that *E. faecium* isolate X3877 carried diverse MRGs including those that represent higher environmental health challenges such as arsenic (*arsA*). For this reason, there is great debate in the EU about the banning of metals in the feed of pig farming to avoid these processes of co-selection to clinically relevant antibiotics [42]. The presence of these MGRs in X3877 of a nestling of a parent stork that forages in a landfill is an indication that metal pollution in the environment is linked to high anthropogenic activities [42, 43].

Virulence factors are very relevant in the pathogenesis of enterococci. Many of these genes such as the *ebpA* seem to be related to clinical enterococcal strains [18, 44]. Other virulence genes that contribute to the colonization and persistence of enterococcal infections were also identified in most of the isolates [45, 46]. Also, the *optrA*-carrying *E. faecalis* isolates recovered from dog, pigs and pig farmers carried a*ce*, *gelE* and *agg* genes, previously described in hospital-associated *E. faecalis* isolates [35, 47].

Regarding virulence factors of the *poxtA*-positive *E. faecium* X3877 isolate, it carried the *acm* and *efaA* genes that encode collagen-binding, although it seems the *efaA* is widely distributed in most *E. faecium* isolates, regardless of whether they are clinical or commensal strains [13].

Generally, the *E. faecalis* isolates analyzed carried plasmids belonging to many of the known replicon families in enterococci [35, 48]. The variability of plasmid content found illustrates the diverse nature of MGE in enterococci and their potential to facilitate the dissemination of some critical AMR genes, such as the *optrA*, as in the case of the *E. casseliflavus* isolate. Importantly, four plasmid replicons were found bound to genes that mediate resistance to tetracyclines, aminoglycosides and chloramphenicol. This could explain the reason why these AMR genes (*tet*(M), *tet*(L), *cat* and *ant4’*) persist in the enterococci and other related bacteria (such as staphylococci) from pigs and pig farmers and the farm environments [35, 49].

Oftentimes, the *fexA* gene is associated with Tn*554*, as previously reported in LZD^R^-*E. faecalis* [35, 50]. However, only one *E. faecalis* isolate carried this transposon in our study**.** The *lnuG* gene identified confers resistance to lincomycin through nucleotidylation in enterococci [51, 52]. The detection of *lnuG* in the LZD^R^ *E. faecalis*-ST330 isolates from two pigs could lead to increased dissemination of lincosamide resistance in the food chain through Tn*6260* [52].

Concerning phages in enterococci, when detected, most intact prophage-associated sequences are found in clinical enterococci in humans and in animal populations [53], but their distribution and involvement in the pathogenesis of enterococcal infection are poorly characterized [47]. Nonetheless, it is important to mention that the therapeutic potential of some of the identified intact prophages has previously been evaluated [54, 55].

Contrary to most other *poxtA*-carrying *E. faecium* isolates, the *fexB* gene in our isolate was in entirely a different contig of the genome, which is often downstream of *poxtA* [23, 56]. Perhaps, the termination of the contig carrying the *poxtA* in our *E. faecium* isolate precludes the co-location of the *fexB* on the same contig.

The phylogenomic analyses from this study strongly suggests the zoonotic transmission of LZD^R^-*E. faecalis* isolates and highlight the impact of pigs’ gut bacteria (such as multidrug-resistant *E. faecalis*) contaminating the farm environment and finding its way to the farmer’s nostrils and other body parts [57, 58]. This is a situation of occupational biohazard on the side of the pig farmer [57, 58]. Moreover, the SNP-based phylogeny illustrates the potential flow and transfer of LZD^R^-*E. faecalis* isolates from multiple sources and countries.

## Conclusions

The data presented in this study comprise one of the few available comprehensive genomic datasets of LZD^R^ enterococci in healthy hosts, including key AMR genes, virulence, prophages, and the plasmidome. The phylogenetic relatedness of our *optrA*-positive *E. faecalis* with those of publicly available genomes from discrete lineages but varied sources and geographical locations revealed close relatedness with most strains from Spain, and other European, and American countries. Additionally, our study detected an interesting variability of *optrA* in some *E. faecalis* isolates of the same lineage (ST330). Findings from this study demonstrated the transmission of LZD^R^-*E. faecalis* between a pig and a pig farmer (zoonosis) and highlight the need to strengthen molecular surveillance of LZD^R^ enterococci in all ecological niches to direct appropriate control strategies. A larger number of isolates could have provided more credence to the findings obtained. However, as linezolid resistance is a rare trait in healthy humans and animals, the data from this study enhances our comprehension of the molecular epidemiology of this critical resistance in healthy hosts.

### Supplementary Information

Below is the link to the electronic supplementary material.

Supplementary file1 (XLSX 13 KB)
